# Case Report: Identification of Compound Heterozygous Mutations in a Patient With Late-Onset Glycogen Storage Disease Type II (Pompe Disease)

**DOI:** 10.3389/fneur.2022.839263

**Published:** 2022-03-21

**Authors:** Huiting Zhang, Jun Chen, Yuchang Zhu, Xiaotang Ma, Wangtao Zhong

**Affiliations:** Guangdong Key Laboratory of Age-Related Cardiac and Cerebral Diseases, Department of Neurology, Institute of Neurology, Affiliated Hospital of Guangdong Medical University, Zhanjiang, China

**Keywords:** late-onset Pompe disease, glycogen storage disease type II, c.1411_1414del, acid α-glucosidase enzyme, metabolic myopathy

## Abstract

Pompe disease is an autosomal recessive hereditary lysosomal disorder and correlated with acid α-glucosidase enzyme (GAA) deficiencies, which lead to accumulation of glycogen in all tissues, most notably in skeletal muscles. Adult late-onset Pompe disease (LOPD) is a slowly progressive disease of proximal myopathy with later involvement of the respiratory muscles, resulting in respiratory failure. In this study, we reported a 22-year-old Chinese woman with inability to withstand heavy physical activity since childhood, who presented with respiratory and ambulation weakness in 2 months. On admission, her bilateral upper limbs strength was 4/5 and lower limbs strength was 3/5 according to Medical Research Council (MRC) score. The patient had compound heterozygotes containing a newly identified 4 nt deletion of coding sequence (deletion nt 1411_1414) in one of the acid α-glucosidase alleles and a c.2238G>C (p.Trp746Cys) missense mutation. This deletion has been reported in infant-onset Pompe disease (IOPD) but not LOPD. Intriguingly, this deletion mutation was not found in the patient's family and was considered as pathogenic. Muscle biopsy showed scattered vacuoles with basophilic granules inside the subsarcolemmal area, which were strongly stained by periodic acid-Schiff (PAS). Laboratory tests revealed a significant increase of creatine kinase MB isoenzyme (CK-MB) and lactate dehydrogenase (LDH). GAA level was 9.77 nmol/1 h/mg and was not sufficient for the diagnosis of GAA activity deficiency (0–3.78 nmol/1 h/mg). In summary, mutational analysis of GAA and muscle biopsy are crucial in the diagnosis of Pompe disease.

## Introduction

Pompe disease is an inherited metabolic myopathy (1). It is reported that the frequency of Pompe disease is 1:50,000 in China and 1:40,000 in Caucasian populations (1, 2). Considering its defects in acid α-glucosidase enzyme (GAA) activity, which leads to glycogen accumulation in lysosomes, Pompe disease is also known as glycogen storage disease type II (1).

The diagnosis of Pompe disease could be very difficult since its clinical manifestation is highly variable and routine laboratory tests lack sensitivity. Muscle biopsy, GAA activity test, and genetic analysis of the GAA gene play important roles in the diagnosis of Pompe disease. GAA gene is located on chromosome 17q25.2-q25.3 and contains 20 exons. At present, ~562 mutations have been discovered in GAA (http://www.pompevariantdatabase.nl.) (3). Among them, c.-32-13T>G mutation is common among Caucasian late-onset Pompe disease (LOPD) patients, while it is not found in Asian population (4), and p.R854X mutation is specifically pronounced in African Americans (5). Two variations, p.G576S and p.E689K, are more frequent among Asian population, including the population in Taiwan and Japan (6). These suggest that GAA gene mutation in Pompe disease has regional and ethnic differences. Pompe disease is an autosomal recessive disorder and mainly divided into infant-onset Pompe disease (IOPD) and LOPD. IOPD usually leads to hypertrophic cardiomyopathy. In IOPD, symptoms occur very early (at a median age of 2 months), and death happens soon afterward if the disease remains untreated (by a median age of 8.7 months) (7). As for LOPD, its manifestations are diverse and typically present with ambulatory and respiratory weakness (8). The diagnosis of LOPD is challenging due to very mild clinical presentations or clinical similarities with other muscular diseases. Therefore, GAA sequencing analysis may help screen Pompe disease. In this study, we reported a deletion mutation in GAA gene, which has not been discovered in LOPD.

## Case Report

A 22-year-old Chinese woman was referred to our hospital (Affiliated hospital of Guangdong Medical University, Zhanjiang, Guangdong, China), with progressive dyspnea and ambulation weakness for 2 months, especially with difficulties in walking up and down the stairs. She was first treated in the department of respiratory medicine and then referred to the neurological department for further diagnosis and treatment. Her exercise tolerance had been waning since elementary school. She could take care of her daily activities, but could not bear heavy physical work. Her family history of consanguinity was negative and birth history was unremarkable. Both her parents and her sister were workers and were healthy. She was the youngest of three siblings.

On admission at the neurological department, the patient's height and weight were 156 cm and 36.2 kg, respectively. Physical examination revealed that her chest expansion was poor and tendon reflexes were evidently decreased in both upper and lower extremities. Her speaking and swallowing functions were normal and no muscle atrophy was observed. Six groups of muscles (shoulder abduction, elbow flexion, wrist extension, hip flexion, knee extension, and foot dorsiflexion) of the patient were assessed by Medical Research Council (MRC) score, which scores muscle strength from 0 to 5. The patient's flexor and extensor muscles of wrist and forearm and intrinsic muscles of feet were scored 5/5. Elbow flexion, wrist and finger extension, foot dorsiflexion, and grip strength were normal. Shoulder abduction strength was 4/5, and hip flexion and knee extension strength were 3/5. Laboratory studies revealed elevated levels of creatine kinase MB isoenzyme (CK-MB) of 51.4 IU/L (normal range: 2.0–5.0 IU/L) and lactate dehydrogenase (LDH) of 392.9 U/L (normal range: 89–221 U/L). Creatine kinase, alanine aminotransferase, and aspartate aminotransferase levels were normal. Red blood cell count (RBC) and hemoglobin (HGB) increased to 7.37 × 10^12^/L (normal range: 4.0–5.5/L) and 174.3 g/L (normal range: 110–150 g/L), respectively. Hematocrit (HCT) was also upregulated to 59.8% (normal range: 33.5–45.5%). N-terminal pro-brain natriuretic peptide (NT-proBNP) was 2,038 pg/ml (normal range: 0–300 pg/ml). Rheumatoid factor, C reactive protein, anti-streptolysin O antibodies, erythrocyte sedimentation rate, antinuclear antibody series and antineutrophil cytoplasmic antibody were all negative. Sinus tachycardia, QRS wave right axis deviation, and chest lead clockwise rotation were found by Electrocardiogram (ECG). Echocardiogram and cerebrospinal fluid test were normal.

On the first night of hospitalization in the neurological department, the patient's dyspnea symptom worsened and no dry or wet rales were heard in both lungs. Her heart rate and blood oxygen saturation were 117 beats/min and 97%, respectively. There was no obvious improvement in her symptoms after antiasthmatic, diuretic, and cardiotonic therapy. Additionally, she presented with blurred consciousness, restlessness, and urinary incontinence in short time. Urgent head CT scan showed that multiple overdue strip high-density shadows presented in the sulci of the bilateral cerebral hemispheres, which probably were sulcal blood vessels or subarachnoid hemorrhage (Supplementary Figure 1). Chest CT revealed bilateral pleural effusion and inflammation in the lungs, with more significance on the right (Supplementary Figure 2). Chest X-ray showed an increased cardiothoracic ratio and inflammation in the lungs. Blood gas analysis showed significantly decreased pH value of 7.057 (normal range: 7.35–7.45) and increased level of pCO_2_ of 18.4 kPa (normal range: 4.26–5.99 kPa). Therefore, the patient needed prolonged ventilator support due to decreased inspiratory muscle strength and worsening of lung function.

**Figure 1:**
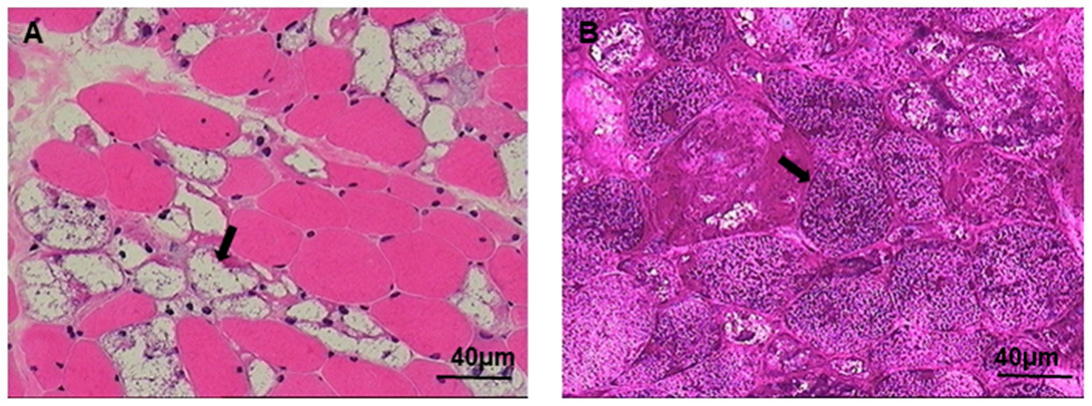
Muscle biopsy is shown using the Hematoxylin and Eosin (HE) and periodic acid-Schiff (PAS) staining. **(A)** Large numbers of glycogen-containing vacuoles were found in the muscle fibers (arrow). **(B)** Vacuoles were PAS positive stained (arrow), scale bar: 40 μm.

**Figure 2:**
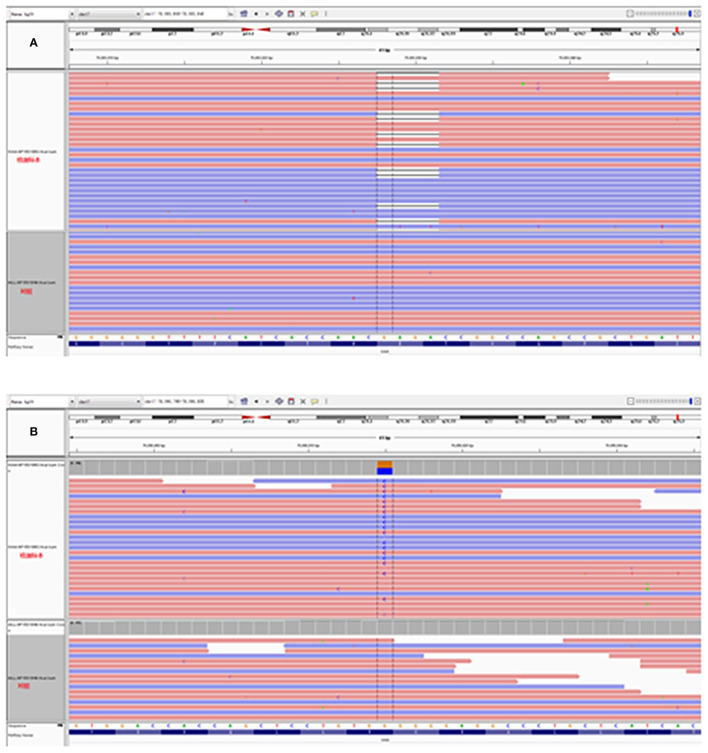
Molecular DNA analysis of the patient. **(A,B)** Heterozygous mutations at c.1411_1414del (p.Glu471ProfsTer5) and c.2238G>C (p.Trp746Cys) were detected in the patient.

**Figure 3:**
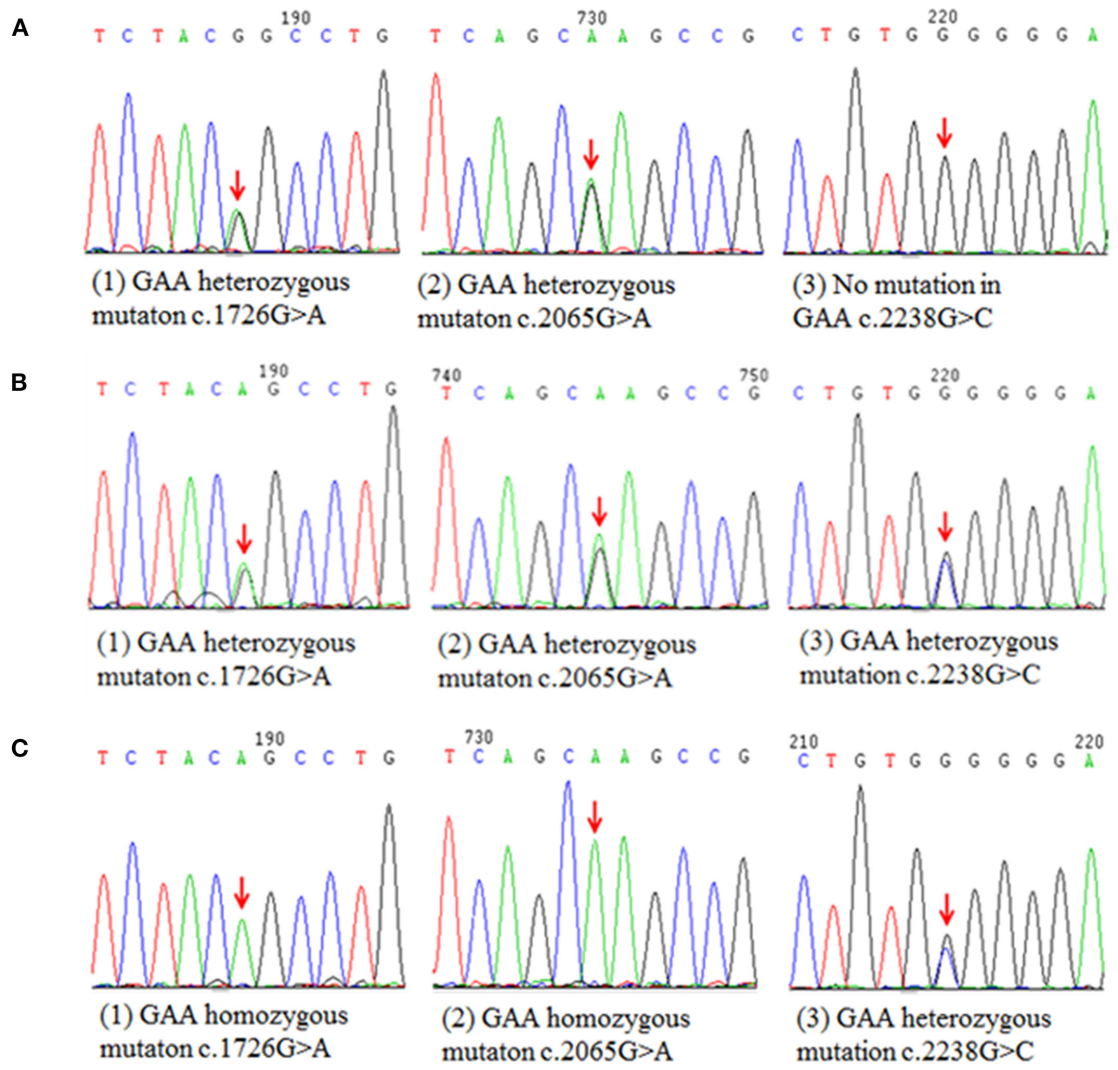
Schematic diagram of the mutation sites of patient's parents and elder sister. **(A)** Two compound heterozygous mutations detected in patient's farther for c.1726G>A (p.Gly576Ser) and c.2065G>A (p.Glu689Lys). **(B)** The patient's mother was heterozygous for c.1726G>A (p.Gly576Ser), c.2065G>A (p.Glu689Lys), and c.2238G>C (p.Trp746Cys) mutations. **(C)** The patient's elder sister was homozygous for c.1726G>A (p.Gly576Ser) and c.2065G>A (p.Glu689Lys) mutations and heterozygous for c.2238G>C (p.Trp746Cys) mutation.

GAA activity of the patient markedly decreased to 9.77 nmol/1 h/mg (normal >14 nmol/1 h/mg), but was not sufficient for the diagnosis of GAA activity deficiency according to the manufacturer instruction (0–3.78 nmol/1 h/mg). We next performed a muscle biopsy of the left quadriceps, after obtaining written consent from the patient. Results showed that plenty of vacuoles were found in the muscle fiber and muscle pulp, and most vacuoles were in the subsarcolemmal area. Basophilic amorphous materials were detected in the scattered intracytoplasmic vacuoles (Figure 1A). Periodic acid-Schiff (PAS) staining disclosed that abnormal glycogen particle deposition, which stained purplish red, were observed in the vacuoles (Figure 1B). Genetic analysis revealed two compound heterozygous mutations at c.1411_1414del (p.Glu471ProfsTer5) in exon 9 and c.2238G>C (p.Trp746Cys) in exon16 (Figures 2A,B) in the patient. The patient's father had two compound heterozygous mutations for c.1726G>A (p.Gly576Ser) and c.2065G>A (p.Glu689Lys) (Figure 3A). The patient's mother was heterozygous for c.1726G>A (p.Gly576Ser), c.2065G>A (p.Glu689Lys), and c.2238G>C (p.Trp746Cys) mutations (Figure 3B), and the patient's elder sister was homozygous for c.1726G>A (p.Gly576Ser) and c.2065G>A (p.Glu689Lys) mutations and heterozygous for c.2238G>C (p.Trp746Cys) mutation (Figure 3C). Nerve conduction, electromyography, and muscle MRI examinations were not performed on the patient as she could not be taken off the ventilator.

The patient showed slight improvement in limb weakness after 1 mg neostigmine intramuscular injection. She was first diagnosed with myasthenia gravis and received Gamma globulin (0.4 g/kg × 5 days) combined with methylprednisolone (500 mg/d × 3 days) treatments. However, there was no improvement in her condition, and later the negative results of acetylcholine receptor (AChR) and muscle-specific tyrosine kinase (MuSK) antibodies in serum helped exclude myasthenia gravis. Therefore, the pyridostigmine bromide and corticosteroids treatments were interrupted. The patient continued to receive anti-inflammatory and mechanical ventilation therapy. She had no access to the recombinant human GAA (rhGAA) treatment because of her poor economic condition. A permanent tracheostomy was performed considering her long-term need of ventilator. Besides, physiotherapy and rehabilitation support were implemented. She was discharged with home ventilator support. Her shoulder abduction strength improved to 5/5 and hip flexion and knee extension strength improved to 4/5 on MRC, and the reflexes and ECG were normal. Six months later, the patient could walk without ventilator support for about 15 min and could get rid of ventilator at rest during daytime, as well as while dressing and showering by herself.

## Discussion

In this study, we reported a patient who presented with adult-onset respiratory and proximal limbs weakness, and compound heterozygous mutation of the GAA gene. Among the heterozygous mutation, c.1411_1414del (p.Glu471ProfsTer5) was a rare and damaging mutation for LOPD. This study expands the clinical and molecular spectrum of LOPD.

Pompe disease, also known as glycogen storage disease type II or acid maltase deficiency, is an infrequent disorder of the glycogen metabolism (1). The diagnosis of Pompe disease could be relatively simple due to the marked severity of clinical symptoms, muscle pathology, and gene mutation analysis. However, diagnosis of LOPD can be very tough because patients may manifest a more heterogeneous phenotype overlapping with other neuromuscular diseases (9). Adults with LOPD typically present with ambulatory and respiratory difficulties, as in the case that we reported (1). At first, without knowing the results of blood gas analysis and the cause of breath shortness, the patient was treated with medium-high flow oxygen. This oxygen concentration could inhibit the excitatory effect of hypoxia-stimulated respiratory center, resulting in the respiratory depression and failure. We applied myasthenia gravis therapy to treat the patient, but failed to improve her condition. The patient's diagnosis was not confirmed until the results of muscle biopsy and GAA genetic tests were available. Owing to weakness of the respiratory and skeletal muscles caused by Pompe disease, the patient progressively developed chronic hypoxemia and type 2 respiratory failure. Therefore, the patient had to depend on a ventilator. The high levels of RBC, HGB, and HCT might be closely related to the chronic hypoxemia. Chronic hypoxemia could also cause pulmonary vasoconstriction and pulmonary hypertension, and further lead to pulmonary heart disease and right heart failure (HF). The patient presented with more obvious right pleural effusion, which probably resulted from right HF. A study pointed out that the increase in systemic venous pressures causing right HF could prevent venous lymphatic drainage or increase hydrostatic pressure in the bronchial veins and the chest wall, eventually resulting in pleural effusion (10). The increased serum levels of CK-MB and LDH indicated cardiac injury in the patient. The suspicious subarachnoid hemorrhage in the patient's brain was a rare but crucial character of Pompe disease. Excessive glycogen accumulation in the smooth muscle cells of the arteries reduces the elasticity and integrity of the vessel walls, making the brain prone to aneurysms, and intraparenchymal hemorrhage (11). Of note, normal GAA activity should not rule out Pompe disease (12), just as in the case that we reported. Sometimes characteristic muscle histopathology findings of lysosomal glycogenosis and autophagic vacuoles may be totally negative, which makes Pompe disease more difficult to be diagnosed (13). Thus, early and precise diagnosis like GAA gene analysis is needed.

Pompe disease is an autosomal recessive disorder in humans caused by mutations in the GAA gene (1), but the phenotypic expression of this disease happens only if both the alleles of the GAA gene carry a pathogenic mutation (14). In our case, the patient had two compound heterozygous mutations at c.1411_1414del (p.Glu471ProfsTer5) in exon 9 and c.2238G>C (p.Trp746Cys) in exon16. The deletion of 1411_1414 causes a reading frameshift after codon 471, a premature termination signal 12 nucleotides downstream of the deletion and a truncated protein of 474 amino acids. This truncated protein lacks the catalytic site of acid alpha-D-glucosidase, localized to codons 516–520 (15, 16). As far as we acknowledge, c.1411_1414del heterozygous mutation in GAA gene has only been reported in IOPD (16, 17). The second mutation at c.2238G>C causes a change from non-polar aromatic tryptophan to polar aliphatic cysteine at codon 746 and has been known to affect the enzymatic function of acid. One study pointed out that c.2238G>C is the most common mutation among Chinese patients with LOPD (18). In addition, according to the Pompe disease database on http://www.pompecenter.nl, the effect of c.1411_1414del (p.Glu471ProfsTer5) mutation is very severe, while 2238G>C mutation is potentially mild. In other words, the patient carried two potentially pathogenic c.1411_1414del (p.Glu471ProfsTer5) and c.2238G>C (p.Trp746Cys) mutations, and the mutation of c.1411_1414del (p.Glu471ProfsTer5) was likely a more severe pathogenic mutation.

The patient's families had no symptoms of Pompe disease and all carried c.1726G>A (p.Gly576Ser) and c.2065G>A (p.Glu689Lys) common mutations. These two mutations are pseudodeficiency mutations with a high carrying rate in the population (19). Approximately 3.9% of Asians carry c.1726G>A and c.2065G>A homozygous mutations, which lead to the decreased number and activity of the enzyme without causing any clinical symptom (20, 21). The patient's parents and sister were, respectively, heterozygous and homozygous for these two mutations, and her mother and sister had an extra heterozygous mutation of c.2238G>C (p.Trp746Cys), which is a missense mutation (22). Previous studies have demonstrated that the pseudodeficiency mutation of c.2238G>C, c.1726G>A, and c.2065G>A slightly or hardly contribute to Pompe disease (19, 21). Overall, according to the genetic analysis of the patient and her families, the c.1411_1414del (p.Glu471ProfsTer5) mutation is a *de novo* mutation. We theorized that the heterozygous mutations of c.2238G>C (p.Trp746Cys) and c.1411_1414del (p.Glu471ProfsTer5) contributed to Pompe disease. To our knowledge, it is the first time that this deletion mutation was found in LOPD.

## Conclusion

In this paper, we reported an LOPD patient who manifested with ambulatory and respiratory difficulties, high plasma levels of CK-MB and LDH, glycogen-containing vacuoles of muscle biopsy, and a rare c.1411_1414del (p.Glu471ProfsTer5) mutation. We emphasized the importance of muscle biopsy and GAA gene analysis in diagnosing respiratory and ambulatory weakness. With the finding of this deletion mutation, the genotypic spectrum of Chinese LOPD patients could be extended. Further investigation and analysis of brain magnetic resonance image (MRI), MR venography, MR angiography (MRA), and muscle MRI could provide more insightful understanding of Pompe disease. Medium-high flow oxygen therapy probably could exacerbate respiratory depression/failure without knowing the results of blood gas analysis and the cause of respiratory weakness.

## Data Availability Statement

The datasets presented in this article are not readily available due to ethical and privacy restrictions. Requests to access the datasets should be directed to the corresponding author.

## Ethics Statement

The studies involving human participants were reviewed and approved by Ethics Committee of Affiliated Hospital of Guangdong Medical University (Permitted Number: PJ2021-094). The patients/participants provided their written informed consent to participate in this study. Written informed consent was obtained from the individual(s) for the publication of any potentially identifiable images or data included in this article.

## Author Contributions

HZ, JC, and WZ: conception and design. JC and YZ: performance of experiments. HZ and WZ: manuscript writing. XM and WZ: review, revision, correction of the manuscript, and study supervision. HZ and JC: data analysis. All authors contributed to the article and approved the submitted version.

## Funding

This study was supported by National Natural Science Foundation of China (81870580), Guangdong Medical Research Foundation (B2018048), Science and Technology Research Project of Zhanjiang City (2018B01012), and Doctor Foundation of Affiliated Hospital of Guangdong Medical University (2021023562).

## Conflict of Interest

The authors declare that the research was conducted in the absence of any commercial or financial relationships that could be construed as a potential conflict of interest.

## Publisher's Note

All claims expressed in this article are solely those of the authors and do not necessarily represent those of their affiliated organizations, or those of the publisher, the editors and the reviewers. Any product that may be evaluated in this article, or claim that may be made by its manufacturer, is not guaranteed or endorsed by the publisher.

## Supplementary Material

Supplementary Figure 1 Brain computed tomography (CT) images of the patient. Brain CT scan showed scattered high-density areas in bilateral cerebral sulci (red arrows).

Supplementary Figure 2 Chest CT images of the patient. Chest CT scan showed bilateral pleural effusion and inflammation in the lungs, which was more obvious in the right lung.

Supplementary Figure 3 Clinical presentation of the patient. **(A)** The patient relied on mechanical ventilation therapy during hospitalization. **(B,C)** No significant muscle atrophy was seen in the patient and she could depend on home ventilator support on discharge.

## References

[1] Van der Ploeg AT Reuser AJ. Pompe's disease. Lancet. (2008) 372:1342–53. 10.1016/S0140-6736(08)61555-X 18929906

[2] De Groot AS Desai AK Lelias S Miah SMS Terry FE Khan S. Immune tolerance-adjusted personalized immunogenicity prediction for Pompe Disease. Front Immunol. (2021) 12:636731. 10.3389/fimmu.2021.636731 34220802 PMC8242953

[3] Niño MY In't Groen SLM Bergsma AJ van der Beek NAME Kroos M Hoogeveen-Westerveld M. Extension of the Pompe mutation database by linking disease-associated variants to clinical severity. Hum Mutat. (2019) 40:1954–67. 10.1002/humu.23854 31254424 PMC6851659

[4] Er TK Chen CC Chien YH Liang WC Kan TM Jong YJ. Development of a feasible assay for the detection of GAA mutations in patients with Pompe disease. Clin Chim Acta. (2014) 429:18–25. 10.1016/j.cca.2013.10.013 24444888

[5] Cheng YS Li R Baskfield A Beers J Zou J Liu C. A human induced pluripotent stem cell line (TRNDi007-B) from an infantile onset Pompe patient carrying p.R854X mutation in the GAA gene. Stem Cell Res. (2019) 37:101435. 10.1016/j.scr.2019.101435 31026687 PMC6658133

[6] Kroos MA Mullaart RA Van Vliet L Pomponio RJ Amartino H Kolodny EH. p.[G576S; E689K]: pathogenic combination or polymorphism in Pompe disease? Eur J Hum Genet. (2008) 16:875–9. 10.1038/ejhg.2008.34 18301443

[7] Kishnani PS Hwu WL Mandel H Nicolino M Yong F Corzo D. A retrospective, multinational, multicenter study on the natural history of infantile-onset Pompe disease. J Pediatr. (2006) 148:671–6. 10.1016/j.jpeds.2005.11.033 16737883

[8] Moriggi M Capitanio D Torretta E Barbacini P Bragato C Sartori P. Muscle proteomic profile before and after enzyme replacement therapy in late-onset Pompe Disease. Int J Mol Sci. (2021) 22:2850. 10.3390/ijms22062850 33799647 PMC8001152

[9] Jones HN Hobson-Webb LD Kuchibhatla M Crisp KD Whyte-Rayson A Batten MT. Tongue weakness and atrophy differentiates late-onset Pompe disease from other forms of acquired/hereditary myopathy. Mol Genet Metab. (2021) 133:261–8. 10.1016/j.ymgme.2021.05.005 34053870

[10] Ferreiro L Álvarez-Dobaño JM Valdés L. Can right heart failure cause pleural effusion? Arch Bronconeumol. (2019) 55:453–4. 10.1016/j.arbr.2019.02.008 30971367

[11] Mormina E Musumeci O Tessitore A Ciranni A Tavilla G Pitrone A. Intracranial aneurysm management in patients with late-onset Pompe disease (LOPD). Neurol Sci. (2021) 42:2411–9. 10.1007/s10072-020-04819-2 33067680

[12] Kishnani PS Steiner RD Bali D Berger K Byrne BJ Case LE. Pompe disease diagnosis and management guideline. Genet Med. (2006) 8:267–88. 10.1097/01.gim.0000218152.87434.f3 16702877 PMC3110959

[13] Chan J Desai AK Kazi ZB Corey K Austin S Hobson-Webb LD. The emerging phenotype of late-onset Pompe disease: a systematic literature review. Mol Genet Metab. (2017) 120:163–72. 10.1016/j.ymgme.2016.12.004 28185884

[14] Muraoka T Murao K Imachi H Kikuchi F Yoshimoto T Iwama H. Novel mutations in the gene encoding acid α-1,4-glucosidase in a patient with late-onset glycogen storage disease type II (Pompe disease) with impaired intelligence. Intern Med. (2011) 50:2987–91. 10.2169/internalmedicine.50.5563 22185990

[15] Hoefsloot LH Hoogeveen-Westerveld M Kroos MA van Beeumen J Reuser AJ Oostra BA. Primary structure and processing of lysosomal alpha-glucosidase; homology with the intestinal sucrase-isomaltase complex. EMBO J. (1988) 7:1697–704. 10.1002/j.1460-2075.1988.tb02998.x 3049072 PMC457155

[16] Shieh JJ Lin CY. Identification of a small deletion in one allele of patients with infantile form of glycogen storage disease type II. Biochem Biophys Res Commun. (1996) 219:322–6. 10.1006/bbrc.1996.0231 8604985

[17] Wan L Lee CC Hsu CM Hwu WL Yang CC Tsai CH. Identification of eight novel mutations of the acid alpha-glucosidase gene causing the infantile or juvenile form of glycogen storage disease type II. J Neurol. (2008) 255:831–8. 10.1007/s00415-008-0714-0 18458862

[18] Zhao Y Wang Z Lu J Gu X Huang Y Qiu Z. Characteristics of Pompe disease in China: a report from the Pompe registry. Orphanet J Rare Dis. (2019) 14:78. 10.1186/s13023-019-1054-0 30943998 PMC6448270

[19] Tajima Y Matsuzawa F Aikawa S Okumiya T Yoshimizu M Tsukimura T. Structural and biochemical studies on Pompe disease and a “pseudodeficiency of acid alpha-glucosidase”. J Hum Genet. (2007) 52:898–906. 10.1007/s10038-007-0191-9 17805474

[20] Taverna S Cammarata G Colomba P Sciarrino S Zizzo C Francofonte D. Pompe disease: pathogenesis, molecular genetics and diagnosis. Aging. (2020) 12:15856–74. 10.18632/aging.103794 32745073 PMC7467391

[21] Kumamoto S Katafuchi T Nakamura K Endo F Oda E Okuyama T. High frequency of acid alpha-glucosidase pseudodeficiency complicates newborn screening for glycogen storage disease type II in the Japanese population. Mol Genet Metab. (2009) 9:190–5. 10.1016/j.ymgme.2009.03.004 19362502

[22] Jia X Shao L Liu C Chen T Peng L Cao Y. GAA compound heterozygous mutations associated with autophagic impairment cause cerebral infarction in Pompe disease. Aging. (2020) 12:4268–82. 10.18632/aging.102879 32126021 PMC7093195

