# Supplementary figures and images for: Case Report: Identification of Compound Heterozygous Mutations in a Patient With Late-Onset Glycogen Storage Disease Type II (Pompe Disease)
Source: Front Neurol. 2022 Mar 21;13:839263. doi: 10.3389/fneur.2022.839263 (PMC8977516; doi:10.3389/fneur.2022.839263)

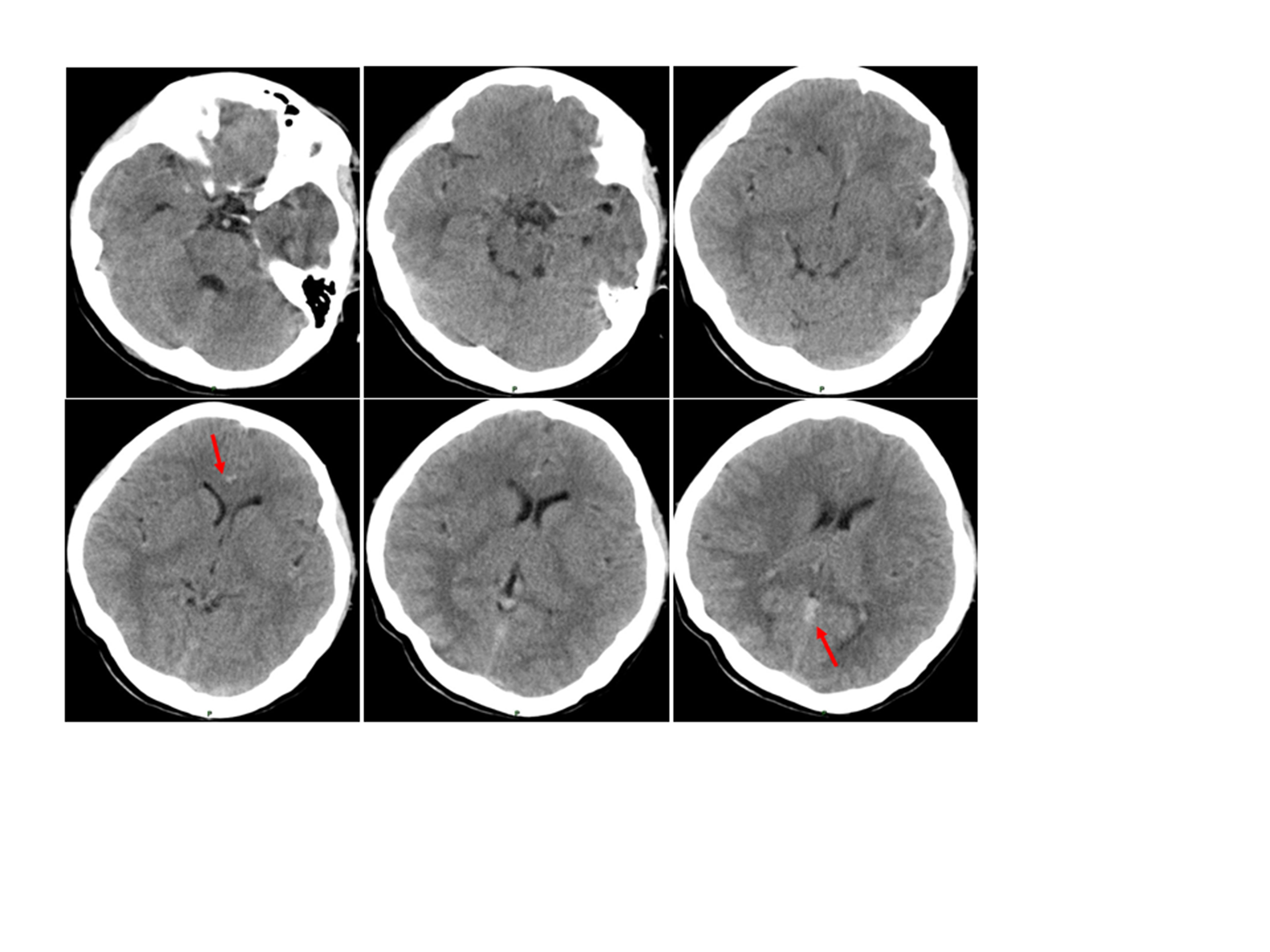

Supplement: Supplementary Figure 1 — Brain computed tomography (CT) images of the patient. Brain CT scan showed scattered high-density areas in bilateral cerebral sulci (red arrows). [file Image_1.TIF]

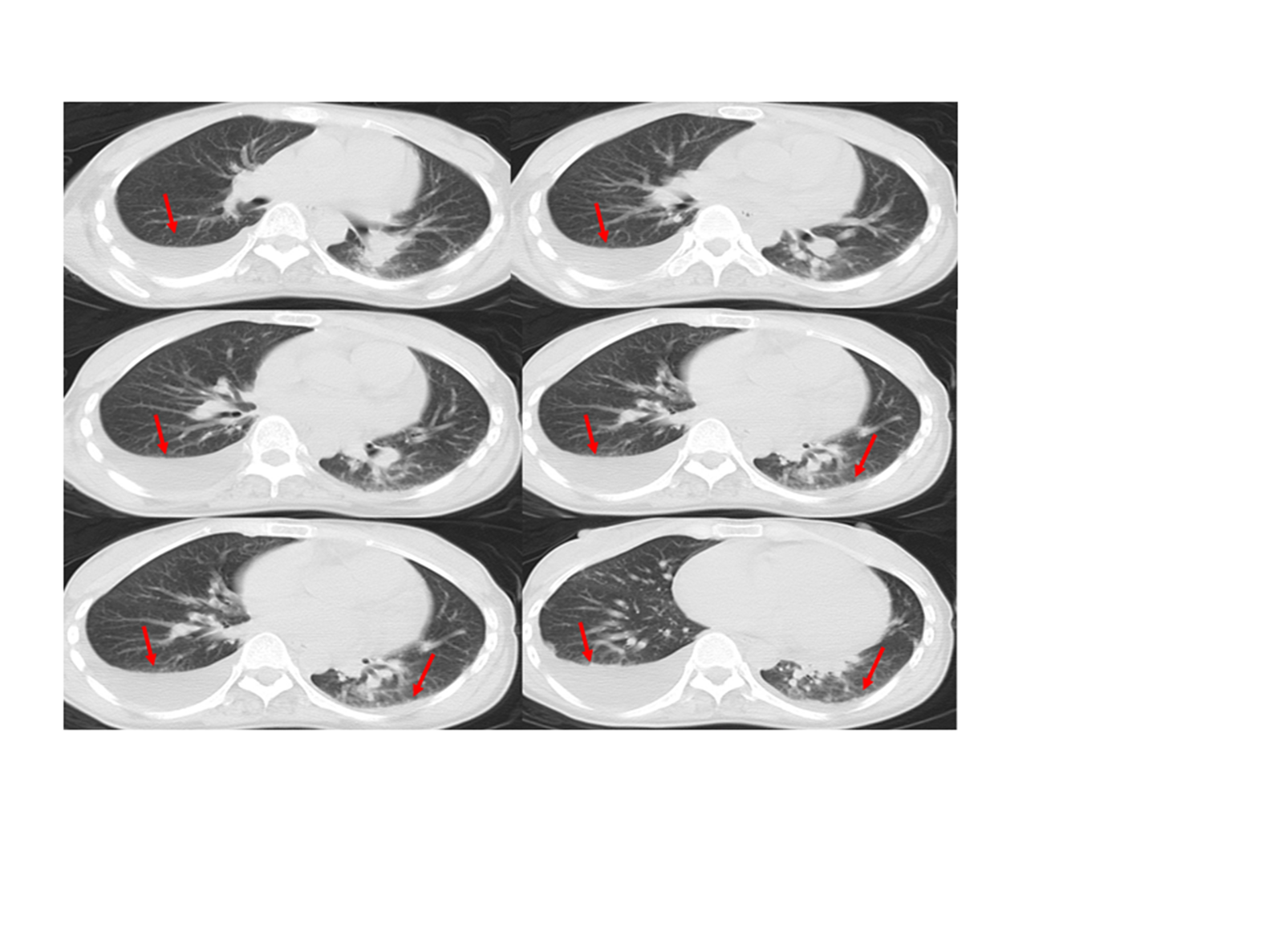

Supplement: Supplementary Figure 2 — Chest CT images of the patient. Chest CT scan showed bilateral pleural effusion and inflammation in the lungs, which was more obvious in the right lung. [file Image_2.TIF]

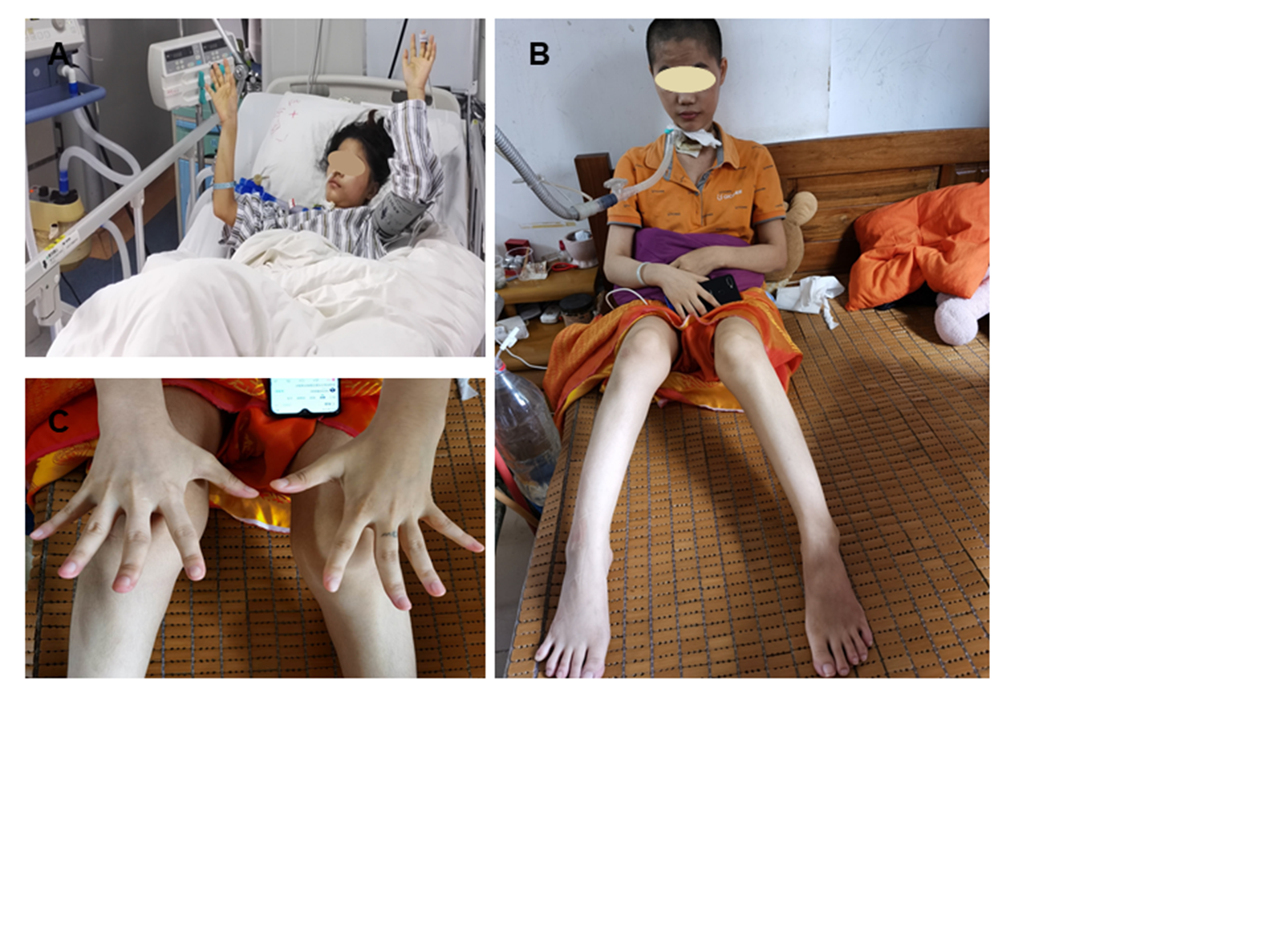

Supplement: Supplementary Figure 3 — Clinical presentation of the patient. (A) The patient relied on mechanical ventilation therapy during hospitalization. (B,C) No significant muscle atrophy was seen in the patient and she could depend on home ventilator support on discharge. [file Image_3.TIF]
